# Spatio-temporal patterns of distribution of West Nile virus vectors in eastern Piedmont Region, Italy

**DOI:** 10.1186/1756-3305-4-230

**Published:** 2011-12-09

**Authors:** Donal Bisanzio, Mario Giacobini, Luigi Bertolotti, Andrea Mosca, Luca Balbo, Uriel Kitron, Gonzalo M Vazquez-Prokopec

**Affiliations:** 1 Department of Animal Production, Epidemiology and Ecology, Faculty of Veterinary Medicine, University of Torino, Italy; 2 Molecular Biotechnology Center (MBC), University of Torino, Italy; 3 Deptartment of Environmental Studies, Emory University, Atlanta, GA, USA; 4 Istututo per le Piante da Legno e l'Ambiente (IPLA), regional government-owned corporation of Regione Piemonte, Torino, Italy; 5 Fogarty International Center, National Institutes of Health. Bethesda, MD, USA

## Abstract

**Background:**

West Nile Virus (WNV) transmission in Italy was first reported in 1998 as an equine outbreak near the swamps of Padule di Fucecchio, Tuscany. No other cases were identified during the following decade until 2008, when horse and human outbreaks were reported in Emilia Romagna, North Italy. Since then, WNV outbreaks have occurred annually, spreading from their initial northern foci throughout the country. Following the outbreak in 1998 the Italian public health authority defined a surveillance plan to detect WNV circulation in birds, horses and mosquitoes. By applying spatial statistical analysis (spatial point pattern analysis) and models (Bayesian GLMM models) to a longitudinal dataset on the abundance of the three putative WNV vectors [*Ochlerotatus caspius *(Pallas 1771), *Culex pipiens *(Linnaeus 1758) and *Culex modestus *(Ficalbi 1890)] in eastern Piedmont, we quantified their abundance and distribution in space and time and generated prediction maps outlining the areas with the highest vector productivity and potential for WNV introduction and amplification.

**Results:**

The highest abundance and significant spatial clusters of *Oc. caspius *and *Cx. modestus *were in proximity to rice fields, and for *Cx. pipiens*, in proximity to highly populated urban areas. The GLMM model showed the importance of weather conditions and environmental factors in predicting mosquito abundance. Distance from the preferential breeding sites and elevation were negatively associated with the number of collected mosquitoes. The Normalized Difference Vegetation Index (NDVI) was positively correlated with mosquito abundance in rice fields (*Oc. caspius *and *Cx. modestus*). Based on the best models, we developed prediction maps for the year 2010 outlining the areas where high abundance of vectors could favour the introduction and amplification of WNV.

**Conclusions:**

Our findings provide useful information for surveillance activities aiming to identify locations where the potential for WNV introduction and local transmission are highest. Such information can be used by vector control offices to stratify control interventions in areas prone to the invasion of WNV and other mosquito-transmitted pathogens.

## Background

West Nile virus (WNV) is a mosquito-borne virus (family *Flaviviridae*) and a neuropathogen for humans, horses, and birds [1]. The virus is indigenous to the Old World, and is maintained in a bird-mosquito transmission cycle primarily involving *Culex *spp. mosquitoes. Humans, horses and other mammals are dead-end hosts for the virus [2]. Neurological human manifestations (< 1% of all cases) are associated with severe morbidity and can be fatal [3]. Early reports of WNV human infections date from the late 1930's in Africa [4]. However, a dramatic expansion of the virus was registered in recent decades, with infections reported in Asia, Europe, and, since 1999, in the New World [5,6]. Early reports of human and equine WNV infection in Europe date from 1964 in the Camargue region, France [7]. Since then the virus has spread throughout Europe, showing an erratic temporal and spatial pattern [8].

Understanding the interrelated ecology of vectors, suitable habitats, and preferential hosts is paramount to predict the emergence and amplification of WNV infection [3]. Multiple European mosquito species are considered competent vectors of WNV: *Culex pipiens *(Linnaeus 1758), *Culex theileri *(Theobald 1903), *Culex modestus *(Ficalbi 1890), *Culex univittatus *(Theobald 1901), *Ochlerotatus caspius *(Pallas 1771), and *Anopheles maculipennis *s.l. (Meighen 1818) [5]. However, only the first three species appear to play an important role as putative vectors in European countries [7,9,10]. In the Volgograd region, Russia, *Cx. pipiens *was involved in WNV transmission in urban areas, and *Cx. modestus *in periurban areas [11]. In France, *Cx. modestus *and *Oc. caspius *are considered the main vectors in wetland areas, and *Cx. pipiens *in urban and periurban areas [12]. In Portugal, the presence of *Cx. pipiens*, *An. maculipennis *s.l., and *Cx. theileri *in wetland areas was associated with two human WNV cases [13].

In Italy, the first WNV cases were recorded in 1998 in a wetland area in the central Tuscany Region (an outbreak involving 14 horses) [14]. After the 1998 outbreak, Italian public health authorities initiated a national surveillance plan aiming to quantify vector abundance, perform targeted vector control in highly infested areas and detect the circulation of WNV. In 1999, *Cx. pipiens*, *Cx. impudicus *(Ficalbi 1890), *Oc. caspius *and *An. maculipennis *s.l. mosquito pools from Tuscany were tested for the presence of WNV infection with negative results [15] . WNV was assumed to be absent in Italy until 2007, when high antibody titers were reported in sentinel birds in the northern Region of Trentino-Alto Adige [16]. In 2008, the largest WNV outbreak --involving 794 horses (from 251 farms) and 9 human infections-- occurred in eight provinces in the northern Regions of Emilia Romagna, Veneto, and Lombardy [10,17,18]. Most cases were located near natural and artificial water sources such as the Po river delta, an area that provides favourable habitats for local and migratory birds [19]. Three pools of *Cx. pipiens *and four of *Oc. caspius *from traps located in the outbreak area were positive for WNV, representing the first isolation of the virus from Italian mosquitoes [10]. WNV transmission continued in 2009, when another outbreak (involving 18 humans and 221 horses, resulting in 4 and 9 fatalities, respectively) occurred in the same area and expanded to the central Italian Regions of Tuscany and Lazio [5,20,21]. In 2010, new equine cases were reported at the end of the summer in the Italian territory: 46 in Sicily, 16 in Molise, 3 in Veneto, and 1 in Emilia Romagna (5 fatal in total) [22], and 13 WNV positive mosquito pools were found in Emilia Romagna and in Veneto Regions [22]. In the same year a jay (*Garrulus glandarius*), a magpie (*Pica pica*) in Emilia Romagna, and sentinels birds in Puglia and Molise (southern Italy) tested positive for WNV [22]. WNV appears to have expanded its range and colonized new areas throughout the country [23], underlining the need for detailed studies determining the suitability of areas not yet invaded for potential establishment of WNV.

In this study we evaluated the effects of environmental (e.g., landscape and weather) determinants on the spatial distribution of *Oc. caspius*, *Cx. pipiens *and *Cx. modestus*. By applying rigorous statistical analysis of longitudinal (2000-2006) CO_2_-baited trap collection data for each of the three putative WNV vectors in the eastern Piedmont Region, Italy, we: (a) described their abundance and spatial distribution, and (b) predicted their geographic distribution based on environmental and ecological data. Our predicted vector distribution maps allowed us to identify areas with high potential risk of WNV introduction and amplification.

## Methods

### Study area

**Figure 1:**
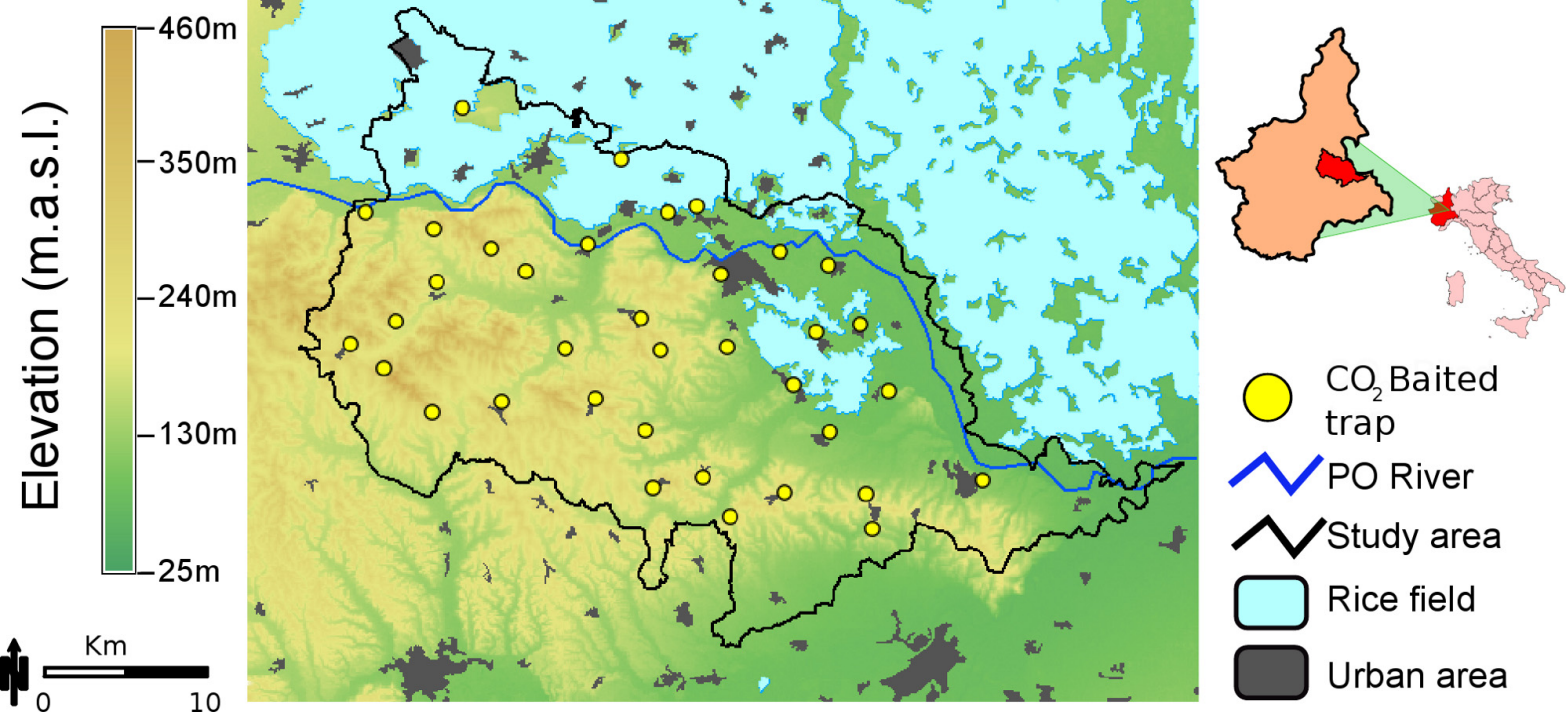
**Map showing the location of the 36 mosquito trapping locations together with topographic and environmental attributes of the study area**. Inset shows the location of the study area within the Piedmont region and Northern Italy.

Our study area covered a territory of 987 km^2 ^in the eastern Piedmont Region (population: 120.593 habitants, centroid: 45.07° N, 8.39° E), North-western Italy (Figure 1). The unique topography and landscape characteristics of this territory are of particular interest for the study of the distribution of potential WNV vectors due to the presence of suitable habitats for migratory birds and abundant larval breeding sites for local mosquito species. The territory (political subdivision equivalent to a US County) is divided evenly between hills (mean elevation 268 m) and plains where the landscape is dominated by mixed agricultural patches (72.2%, mostly in the north-eastern plans), rice fields (14.2%, mostly in northern plans), deciduous tree forests (8.6%, mostly on southern hills), urban environments (3.1%, highly populated cities are located in the plains), and the Po river with its tributaries (1.9%, North) (Figure 1). Cold winters and hot-warm summers (0.4°C and 24.0°C average daily temperature, respectively) and abundant spring and autumn rainfall (~600 mm/yr) are characteristic of the region (Agenzia Regionale per la Protezione dell'Ambiente del Piemonte, ARPA [29]).

### Mosquito surveillance and control

The mosquito population surveillance in the Piedmont programme started in 1997 by Istituto per le Piante da Legno e l'Ambiente (IPLA). Surveillance is constituted by an array of 36 mosquito sampling locations distributed at a minimum distance of 5 km from each other following a random pattern (Figure 1). Trap placement was based on habitat features suitable for mosquito development: near rice fields, woodlands, and at the periphery of urban areas. In each sampling location, weekly overnight adult mosquito collections were performed using CDC CO_2_-baited traps from May to October (the period when mosquitoes are active). After collection, all adult mosquitoes were sexed, counted and identified using proper keys [24].

Different strategies (ongoing since 1998) were implemented in the study area to reduce mosquito abundance. Starting in the spring, breeding sites were treated with larvicides such as *Bacillus thuringiensis *var. *israelensis *(Bti), Temephos and Diflubenzuron. Bti has no toxic effect on other animals and plants [25], but its field performance is greatly influenced by the presence of organic matter or of solids in the water [26]. Highly polluted breeding sites were treated with Themephos, an organophosphate authorized in EU until 2006, and Diflubenzuron, a benzamide inhibiting the production of chitin. Rice fields, the most important breeding sites of the main nuisance species in the area, were treated in a belt of 23.000 hectares around the main cities by performing space sprays of Bti water suspensions with aircrafts. Since 2007, and in order to cut down the cost of aerial applications, rice growers took charge of treating their rice seeds with Diflubenzuron just before seeding.

### Data management and processing

All geographic data were merged in a Geographic Information System (GIS) based on GRASS system [27] and projected from geographic to planar units (UTM zone 32, datum WGS84). Spatial data used to generate mosquito suitability maps included land surface temperature (LST), Normalized Difference Vegetation Index (NDVI), rainfall, elevation, and land-use. LST and NDVI were derived from the Moderate Resolution Imaging Spectroradiometer (MODIS) satellite (National Aeronautics and Space Administration, NASA [28]).

Daily rainfall records for the period 2000-2006 and for 2010 (the year used to create the mosquito prediction maps) for each sample location were derived from a map interpolation (1 km pixel size) generated using the data collected by five ground level weather stations managed by the regional agency of meteorology (ARPA [29]) and spaced ~10 km apart [27].

LST data consisted of spatially continuous 8-day averages at a spatial resolution of 1 km, and NDVI was based on 16-day averages at a resolution of 250 m. All MODIS images were corrected geometrically using ground control points (GCP) of known coordinates and edited to eliminate atmospheric disturbance allowing comparison among different periods [27]. Elevation data (in meters above sea level, m.a.s.l.) were obtained from the Shuttle Radar Topography Mission dataset (90 m pixel size [28]), and land-use was acquired as raster image from the European Environment Agency (100 m pixel size [30]). We generated raster maps depicting the distance of every pixel in the study area to the nearest urban areas, rice fields, and woodlands by applying the straight line distance interpolation function in GRASS [27]. This function calculates the measured Euclidean distance from every point in the study area to the nearest landscape feature, and represents it as a raster file with spatial resolution of 1 km.

### Statistical analysis

#### Spatial analysis

We applied the Ripley's K-function ($\hat{L}\left(d\right)$) [31] to assess whether the distribution of traps within the study area differed from a random distribution. Significance was evaluated by comparing the observed values with the expected values under the complete spatial randomness assumption based on 999 Monte Carlo permutations [31]. The spatial patterns of *Oc. caspius*, *Cx. pipiens *and *Cx. modestus *abundance per trap were quantified by the Getis $G_{i}^{*}\left(d\right)$ local statistic [32]. To identify spatial patterns in mosquito abundance we grouped and analyzed the data for three study seasons: Spring (1^st ^May - 21^st ^June), Early summer (22^nd ^June - 15^th ^August) and Late Summer (16^th ^August - 15^th ^September). Mosquito abundance was estimated as the median number of mosquitoes per week per study season.

#### Statistical modelling

The association between the abundance of each mosquito species and selected environmental and ecological factors was analyzed using a spatially explicit generalized linear mixed model (GLMM). Briefly, the GLMM is an extension of a classic generalized linear model that accounts for correlated data structures (e.g. clustered or longitudinal data) by including random cluster and/or subject effects [33]. The correlated spatial effect in the model was added by considering the geographic position of each sampling location as a structured spatial random effect. The spatial random effect was modelled as a Gaussian Markov random Field [34] using a nearest neighbour structure to define the spatial relationship between sampling locations [35]. We fitted the data using a zero-inflated negative binomial distribution (ZINB) to account for the overdispersion observed in the distribution of the median number of mosquitoes per trap. We used a Bayesian approach based on the Integrated Nested Laplace Approximation (INLA) [34] to fit our GLMM models. The choice of this method was based on its time and analytical efficiency in approximating to the posterior marginal probabilities in comparison to classic MCMC approaches [36].

#### Model parameters

A subset of 30 trap locations with weekly entomologic and environmental information for the period 2000-2006 was used to build the GLMM models (i.e., training dataset), whereas the remaining 6 trap locations were used to test the performance of the models (i.e., test dataset). The period 2000-2006 was chosen because: a) was the time with the highest spatial coverage of CO_2_baited-traps; b) vector control actions in the study region were minimum; and c) from 2007 to 2010 control interventions increased in intensity and quality, potentially impacting our ability to predict the abundance and spatial distribution of each mosquito species.

By selecting environmental and ecological parameters deemed as the most influential in predicting the abundance and spatial distribution of each mosquito species, we outlined the following full model (full code in Additional file 1: Table 2A):

$$\begin{array}{llll} y= & \text{Intercept}+\beta_{\text{1}}\text{*}RAIN+\beta_{\text{2}}\text{*}TWEEK+\beta_{\text{3}}\text{*}SIN+\beta_{\text{4}}\text{* }ELEV+\beta_{\text{5}}\text{*}NDVI\; & & \;\\ & +\beta_{\text{6}}\text{*}DISTU+\beta_{\text{7}}\text{*}DISTR+\beta_{\text{8}}\text{*}DISTW+\beta_{\text{9}}\text{*}RICEA+\beta_{\text{1}0}\text{*}RAIN\text{*}TWEEK\; & & \;\\ & +\beta_{\text{11}}\text{*}RAIN\text{*}NDVI+\beta_{\text{12}}\text{*}TWEEK\text{*}NDVI+RNDtrap+SRNDTRAP\; & & \;\\ & \; & \end{array}$$

Where *y *represented the abundance of each species (i.e., *Oc. caspius*, *Cx. pipiens*, *Cx. modestus*) at the sampling locations. *RAIN *(rainfall) represented the cumulative rainfall 10-17 days prior to trapping, this lagged variable was chosen to take into account the cumulative effect of rain on mosquito productivity and abundance. *TWEEK *represented the average LST for each sampling location during the 8-15 days prior to trapping. This time lag aimed to capture potential impacts of temperature on mosquito population dynamics (by covering the conditions experienced by the earlier stages of larval development). Given the known seasonal pattern in mosquito abundance, we introduced seasonality (*SIN*) as a sinusoidal curve with a phase of 1 year. The *SIN *value was set to have a peak in the first week of August (when abundance of most mosquito species in the study area presents peaks). Elevation (in m.a.s.l) of each sampling location (*ELEV*) was selected to account for topographic (altitudinal) differences across the study area. Since the study area is in an agricultural zone, and the environmental changes during the year could be influenced by human agricultural activities, we used *NDVI *as a surrogate for environmental (i.e., vegetation) change over time. The potential effects of environmental features on mosquito collections were tested by estimating the distance (in km) of each sampling location to urban areas (*DISTU*), rice fields (*DISTR*), and woodlands (*DISTW*). We included in the models the area covered by rice fields (in Km^2^) located nearest to each sampling location (*RICEA*). We also included the interaction between the environmental factors potentially affecting mosquito development: temperature and rainfall (TWEEK * RAIN), NDVI and rainfall (NDVI * RAIN), and temperature and NDVI (TWEEK * NDVI). We considered each sampling location (*RNDtrap*) as an unstructured spatial random effect. The *RNDtrap *represented the difference between each trap which was not possible to account with the chosen regressor, but which can affect the number of collected mosquitoes. We used a spatially structured random effect modelled with a GMRF with a Gaussian distribution (*SRNDtrap*) to represent the effect of the spatial position of each trap location [35]. As the trap location did not vary across years *ELEV*, *DISTUR*, *DISTR*, *DISTW*, and *RICEA *remained as constant values in the model. The *TWEEK*, *RAIN*, and *NDVI *were not constant due to their seasonal variation during the study period. The treatment was not included in the models because all the sampling locations were inside the treated area and so the various control strategies did not have a significant differential effect. We built and tested 169 GLMM models formulated in according with the ecological characteristics of each mosquito species.

### Model selection

The best model (among all tested models) was chosen using the Deviance Information Criterion (DIC) [34,37]. DIC is one of the most commonly used Bayesian selection criteria to select models based on the need to compromise between goodness of fit and model complexity. This index is useful to confront mixed effect models with fixed and random effects because it estimates the effective number of parameters included in the model [38]. DIC is obtained by the sum of the posterior mean of deviance and the number of effective parameters used in the model [38]. Low DIC values are indicative of models showing the best trade off between model fit and complexity, and presenting a good performance predicting unobserved quantities [38,39]. The Logarithmic Score (LS) was used to test the internal cross-validation of the model and its predictive performance, to calculate the cross-validation one observation is excluded from each step of the validation process and the remaining observations are used to perform the predictive distribution [40]. The negative logarithm of predictive distribution is the LS of a given model. The model with the smallest LS has the highest predictive power [40]. Models showing the lowest DIC were considered to best explain the data, and hence selected to predict the abundance of each mosquito species. In case of models with a difference in DIC less than 5 units the one with the smaller LS was chosen

### Vector suitability map

We computed a highly detailed distribution map (500 meter pixel size) of the abundance of *Oc. caspius*, *Cx. pipiens *and *Cx. modestus *using the best GLMMs for all three species. The model parameters (i.e., *β*) from each significant variable were multiplied by the respective rasterized variable to generate a continuous representation of mosquito abundance. The maps were developed to predict the abundance of the three species in August 2010, when they show their peak of high abundance. The year 2010 was selected for the predictions because we were interested in assessing the abundance and spatial distribution of vectors when WNV was known to circulate in Italy, and because these maps could be used to design vector control interventions for 2011.

### Statistical tools

All analyses were performed using R software [41]. GLMM models were fitted using the INLA package [34]. All the GIS operations and spatial analyses were performed with GRASS GIS software [27].

## Results

### Mosquito collections

**Figure 2:**
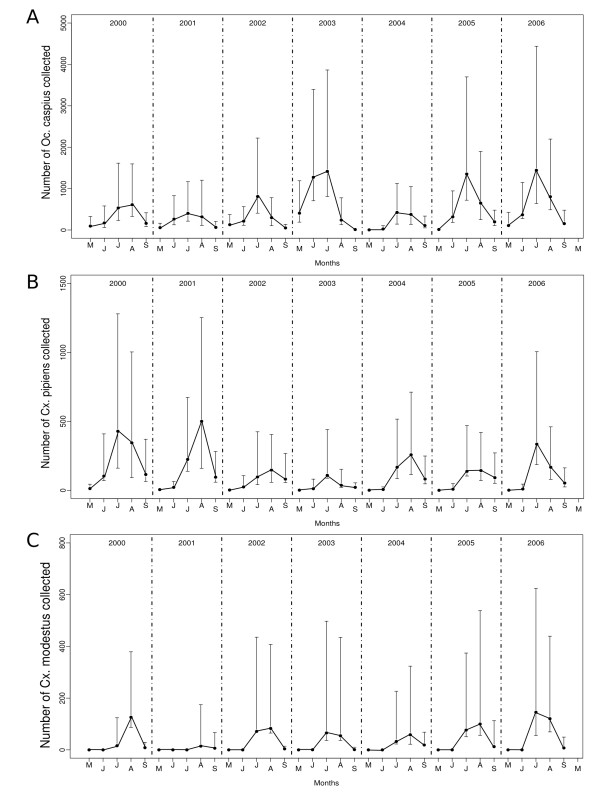
**Median Number of adult mosquitoes collected in 36 CO_2_-baited traps placed in the study area from 2000 to 2006**. (A) *Oc. caspius*, (B) *Cx. pipiens*, and (C) *Cx. modestus*. Values in graphs represent the median (black dots) and interquartile interval (error bars).

A total of 1, 223, 974 female mosquitoes belonging to eleven species were trapped from 2000 to 2006 (Table 1), employing a total trapping effort of 6, 200 trap-nights. The most commonly trapped species were *Oc. caspius *(61.8%), *Cx. pipiens *(21.2%) and *Cx. modestus *(16.3%). All the three species investigated showed a seasonal activity pattern, with abundance peaking in mid-summer (July-August, Figure 2). Monthly *Oc. caspius *adult collections were higher in 2003 (median (Md) = 3, 270, interquartile range (IQR) = 1, 625-4, 420) and 2006 (Md = 3, 522; IQR = 1, 933-5, 516) and lower in 2004 (Md = 596, IQR = 1, 134-1, 598). The highest numbers of *Cx. pipiens *per month were obtained in 2000 with a median (Md = 1, 592; IQR = 621-2, 271) whereas the lowest numbers were obtained in 2003 (Md = 179.5; IQR = 74-402). The minimum number of monthly collected *Cx. modestus *was in 2001 (Md = 36; IQR = 5-273) and increased during 2005 (Md = 266, IQR = 82-816) and 2006 (Md = 343, IQR = 158-1, 105).

**Table 1:** Number of mosquitoes by species collected in 2000-2006 in a 987 km^2 ^area located in eastern Piedmont Region, Italy.

	Sampling year							
**Species**	**2000**	**2001**	**2002**	**2003**	**2004**	**2005**	**2006**	**Tot**.

*Ochlerotatus caspius*	97, 523	70, 276	74, 828	167, 887	51, 898	128, 765	164, 796	755, 973
*Culex pipiens*	75, 835	46, 584	27, 157	16, 842	30, 241	25, 908	37, 452	260, 019
*Culex modestus*	25, 517	16, 662	28, 154	29, 418	25, 065	38, 081	36, 618	199, 515
*Anopheles maculipennis*	1, 972	342	332	878	573	1, 977	1, 722	7, 796
*Anopheles plumbeus*	29	8	75	12	29	8	16	177
*Aedes vexans*	9	23	31	29	47	28	33	200
*Aedes geniculatus*	9	4	20	12	20	38	8	111
*Culiseta annulata*	5	9	20	0	46	9	4	93
*Culiseta subocrhea*	1	16	5	8	22	3	3	58
*Culiseta longiareolata*	0	0	2	23	5	0	1	31
*Coquillettidia richiardii*	0	0	0	1	0	0	0	1
Total	200, 900	133, 924	130, 624	215, 110	107, 946	194, 817	240, 653	1, 223, 974

**Table 2:** Posterior distributions of the fitted terms of spatial GLMM models applied to the weekly abundance of *Oc.caspius*, *Cx. pipiens*, and *Cx. modestus *by CO_2_-baited trap in eastern Piedmont Region, Italy.

	Mosquito Species		
	
	*Oc. caspius*	*Cx. pipiens*	*Cx. modestus*
Fixed Effects**	*Mean (95% CIs)*	*Mean (95% CIs)*	*Mean (95% CIs)*
Intercept	3.671 (2.342; 4.035)*	4.022 (3.532; 4.567)*	2.671 (1.278; 3.678)*
DISTR	-0.034 (-0.089; 0.042)	-	-0.081 (-0.221; 0.054)
DISTU	-	-0.122 (-0.234; 0.073)	-
ELEV	-0.006 (-0.015; 0.002)	-0.014 (-0.012; -0.006)*	-0.021 (-0.026; -0.020)*
RAIN	0.008 (0.005; 0.01)*	0.018 (0.012; 0.023)*	-
TWEEK	0.092 (0.085; 0.112)*	0.070 (0.047; 0.081)*	0.083(0.071; 0.121)*
NDVI	0.798 (0.441; 1.131)*	-	0.864 (0.491; 1.378)*
SIN	0.853 (0.809; 0.942)*	0.278 (0.160; 0.354)*	0.589 (0.341; 0.730)*
*Random Effect*	*Mean (sd)*	*Mean (sd)*	*Mean (sd)*
RNDTRAP	0.234 (0.09)	0.589 (0.141)	0.454 (0.08)
			

* P < 0.05.

** DISTR = distance from rice fields (Km); DISTU = distance from urban area (KM); ELEV = elevation of trapping location (m.a.s.l.); RAIN = cumulative rainfall (mm) for the 10 days before the collection; TWEEK = weekly mean temperature for the week before the sampling (C°); NDVI = NDVI value of the pixel of trapping location; SIN = sinusoidal function representing the seasonality (1 = 1^st ^week of August, 1 year phase); RNDTRAP = unstructured random effect for trapping locations.

**Figure 3:**
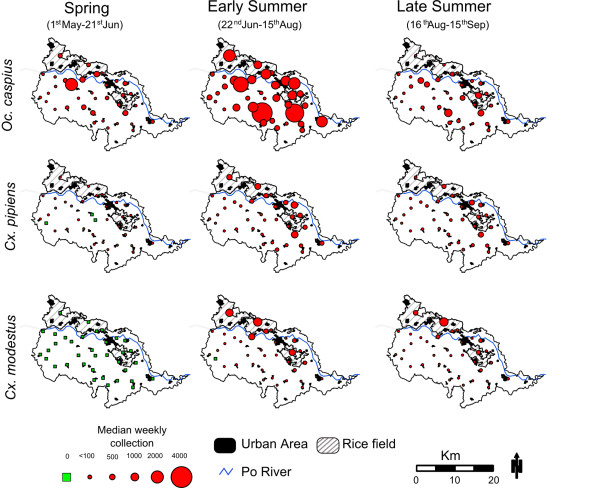
**Spatial pattern of the number of adult mosquitoes collected, by species, during 2000 to 2006 in the eastern Piedmont Region, Italy**. Median of weekly collection (red circles), and the traps that have median collection equal 0 (green triangles). The urban areas and rice fields were also included.

All species showed the highest abundance (weekly median) in the northern extreme of the study area, within an area composed by rice fields, highly populated urban areas and the Po river (Figure 3). *Oc. caspius *extended its spatial distribution during the early summer (22^nd ^June - 15^th ^August), showing high abundance also at higher elevations (Figure 3). *Cx. modestus *adults were practically absent during the spring (1^st ^May - 21^st ^June) when all sample locations had a Md = 0 (IQR = 0-3) (Figure 3).

Temperature trends fluctuated during the 7-year study period (supplementary Figure 1-A). The warmest year was 2003 (mean = 23°C; CI 95% = 21.6-24.3°C) and the coldest was 2002 (mean = 20.5°C; CI 95% = 20-22.7°C), when most rainfall was also occurred. The driest year was 2003 (122 mm) and the wettest 2002 (512 mm) (Additional file 1: Figure 1-A).

### Spatial analysis

**Figure 4:**
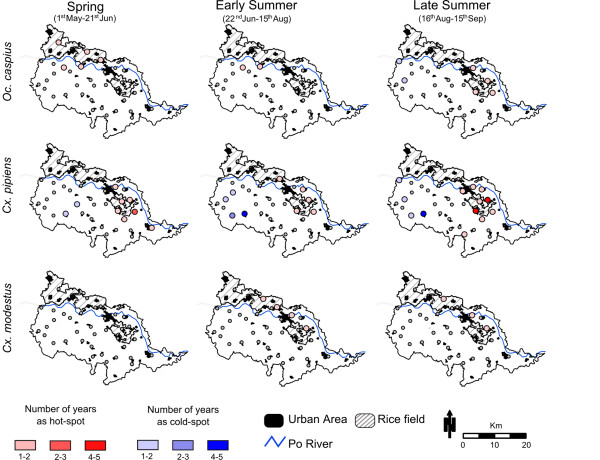
**Occurrence of multiple seasonal spatial clusters of vector abundance during 2000-2006**. The spatial cluster analysis was performed with the $G_{i}^{*}\left(d\right)$ function based on inverse of distance (see methods for more detail). Red scale - hot-spots (clustering of traps with high mosquito abundance). Blue scale - cold-spots (clustering of traps with low mosquito abundance). Urban areas, rice fields, and the Po River were also included.

The seasonal spatial patterns of mosquito distribution showed evidence of clustering (Gi*(d) < 3.71, *P *< 0.05) for the three species tested (Figure 4). Hot- spots of high *Oc. caspius *abundance were recorded in spring and early summer near rice fields in the north-western part of the study area. During late summer, spatial clustering shifted towards the north-east, also near rice fields (Figure 4). *Cx. pipiens *hot-spots were, for all seasons, located in the highly urbanized parts of the study area (i.e., near Casale Monferrato, pop: 36, 095 habitants). This species was the only one that also had cold-spot clusters, which were present in early and late summer in the more rural and hilly parts in the south-west of the study area. The hot-spots of *Cx. modestus *abundance occurred with low intensity (1-2 years) only during summer. They were concentrated near rice fields located mostly in the north-eastern part of the study area (Figure 4). *K-function *analysis confirmed that the observed clustering patterns were not the result of heterogeneous spatial distribution of traps within the study area, as traps were randomly distributed (|*L(d)*| <*d *for all 0 <*d *< 10 Km; *P *> 0.05; data not shown).

### Model results

**Figure 5:**
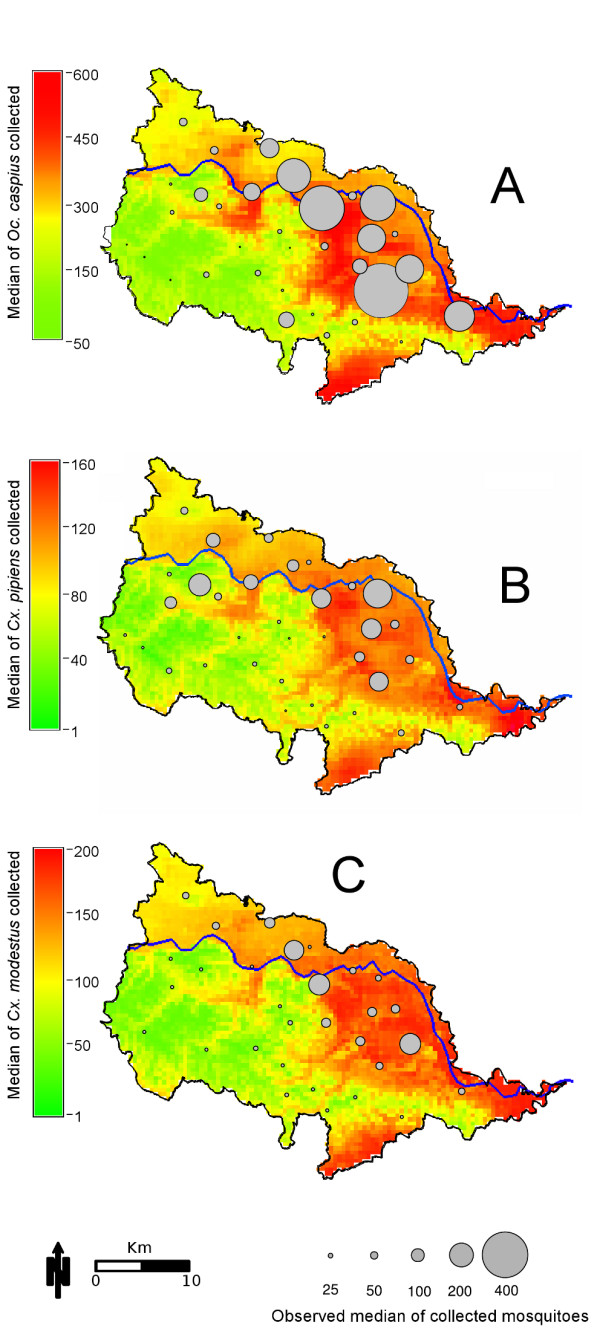
**Prediction maps for *Oc. caspius *(A), *Cx. pipiens *(B) and *Cx. modestus *(C) peak abundance**. Maps were generated by multiplying spatially continuous environmental variables by the regression coefficients from the best GLMM model during the predicted peak vector abundance, summer 2010. The observed data obtained in 2010 were added to the maps. The observed number of collected mosquitoes was expressed using the moving average of the collection performed in August 2010

The DIC and LS of the top three models for *Oc. caspius*, *Cx. pipiens *and *Cx. modestus *are shown in supplementary Table 1-A. The difference in DIC of the top three models for each mosquito species was above 10 units: these differences allow selection of a unique best model for each species. Such models had an LS value closer to zero compared to the other models tested, indicating a high power in predicting the data (Additional file 1: Table 1-A). The parameters estimated for the best GLMM model for each of the three mosquito species tested are summarized in Table 2. The spatial structured random effects were not present in the best model chosen. *Oc. caspius *weekly abundance was significantly and positively associated with the average temperature during the week prior to trapping, cumulative amount of rain during the 10 days prior to trapping, the seasonal fixed effect term (*SIN*), and the bi-weekly NDVI values around a trap (Table 2). Distance to rice fields and elevation, although not statistically significant, had a major influence in determining the best GLMM model for *Oc. caspius. Cx. pipiens *weekly abundance was significantly and positively associated to the cumulative rain during the 10 days prior to trapping, the average temperature during the week prior to trapping, and the seasonal fixed effect term (*SIN*); and negatively associated with the elevation (in m.a.s.l) of each trapping location (Table 2). The inclusion of distance of each trapping location to the nearest urban center was not significant, but had influence in determining the best GLMM model for *Cx. pipiens *(Table 2). The best model of *Cx. modestus *included as significant and positively associated parameters the average temperature during the week prior to trapping, the NDVI and seasonal fixed effect (Table 2). The two negatively associated environmental features were the distance from each trap location to the nearest rice field (non-significant) and the elevation (significant). The best models for the three species showed high predictive power (root mean square error, RMSE, 149.2 ± 129.4, for *Oc. caspius*, 75.3 ± 34.2 for *Cx. pipiens*, and 90.4 ± 18.6 for *Cx. Modestus*), capturing the seasonality (Additional file 1: Figure 3-A, 4-A, 5-A) and spatial distribution (Figure 5) of each species. Residuals plots (Additional file 1: Figure 2-A, 3-A, 4-A, and 5-A) suggest that the best models tended to underestimate the number of collected individuals during the period of peak abundance. The best model of *Cx. modestus *was the only one that overestimated the number of captured adults during the beginning of the sampling season (Additional file 1: Figure 2-A, and 5-A).

### Vector suitability map

The best GLMM models for *Oc. caspius*, *Cx. pipiens *and *Cx. modestus *were used to generate a vector suitability prediction map for the first week of August 2010 (close to the seasonal peak of abundance of each species) (Figure 5). The high abundance of *Oc. caspius *was predicted to occur in close proximity to rice fields present in the northern part of the study area. The predicted distribution of *Cx. pipiens *showed that this species was abundant near the urban areas located in the eastern part of the study area, where the majority of urban centers are located. *Cx. modestus *was predicted to be more abundant in areas where the landscape is dominated by mixed agricultural patches (42%) and rice fields (27%) (North-East and part in the South). The high abundance areas, for all the species, were concentrated within the Po River basin, where rice fields, abundant vegetation and large urban centres may provide unique opportunities of WNV introduction, amplification and transmission. The models accurately predicted the location of traps with high and low vector abundances in August 2010 (Figure 5), validating the ability of GLMM models to predict spatial and temporal patterns of vector abundance in the region.

## Discussion

The impacts of spatially heterogeneous environmental and ecological factors on mosquito population dynamics are complex and poorly understood for many species. Particularly for zoonotic mosquito-borne diseases, understanding the inter-relationships between vectors, hosts, and their environment can provide valuable information for identifying conditions suitable for pathogen introduction, amplification, and transmission. By analyzing a highly detailed longitudinal dataset of the abundance and spatial distribution of the three putative WNV vectors in the eastern Piedmont Region of Italy (i.e., *Oc. caspius*, *Cx. pipiens*, and *Cx. modestus*) we identified a set of environmental and ecological conditions that are favourable for their development, predicted their spatial occurrence, and identified potential areas that, due to their environmental conditions, would be suitable for the introduction and amplification of WNV. The Piedmont Region is located between two European regions where outbreaks of WNV occur annually: Camargue, France, (to the East) and Emilia Romagna, Italy (to the West), increasing the relevance of our findings for the understanding of WNV transmission dynamics in southern Europe.

The three mosquito species varied in degree of heterogeneity of their spatial distribution throughout the study region. Most of the *Oc. caspius *hot-spots occurred near rice fields, where the abundance of larval development habitats and avian hosts allow for very large mosquitoes population [42-44]. Mark-release-recapture experiments have shown that rice fields are not only important breeding habitats for *Oc. caspius *but also serve as major sources of dispersing adults, which were found to be passively dispersed by wind to distances of up to 10 km from their release point [42]. Moreover, the temporal trend in spatial clustering showed differences between hot-spots across seasons: whereas the spring clusters occurred near rice fields located in the North, summer clusters occurred in the North-East, in close proximity to the largest city in the study region (Casale Monferrato). Interestingly, late summer/early fall was the period when most WNV equine cases were reported in Northern Italy [22]. Therefore, clustering of *Oc. caspius *near urban environments during late summer may bring humans into higher risk of exposure to potentially infective mosquito bites.

In the Camargue Region, France, wetlands and rice fields were important hot-spots for the transmission of WNV due to their high vector productivity (particularly *Oc. caspius*, *Cx. pipiens *and *Cx. modestus*) and the occurrence of large equine WNV outbreaks and human cases near by [45,46]. The capacity of *Oc. caspius *to feed interchangeably on birds and mammals makes this species a potential bridge vector for WNV and for other zoonotic arboviruses in proximity to rice fields [12,47]. Moreover, rice fields in the Mediterranean Region are also an important habitat for residential and migratory birds [48], and may represent one of the main entry doors of WNV into Europe.

*Oc. caspius*, *Cx. pipiens*, and *Cx. modestus *may play a key role as enzootic vectors of WNV in eastern Piedmont because they feed both on mammals and birds, but with different preference [11,47,49]. Although laboratory experiments showed a low vector competence of *Oc. caspius *for WNV [12], infected pools of this species have been found recently in Israel and in Italy [10,50], increasing the interest in investigating its role as a potential WNV vector. Given that natural populations of this species can reach very high numbers (being the most important nuisance mosquito species in northern Italy), their potential role as amplifying vectors in eastern Piedmont cannot be ruled out.

*Cx. pipiens *was significantly clustered in the most urbanized areas of the region, the main habitat for this species [51]. This species is the most important WNV vector in USA and one of the main vectors in Europe [3] where it comprises two forms *Cx. pipiens pipiens*, and *Cx. pipiens molestus *[52]. These two sub-species may play a different role in the spill-over of WNV from birds to humans. *Cx. pipiens pipiens *is strictly ornitophilic and feeds only rarely on humans, whereas *Cx. pipiens molestus *is more anthropophilic and may play role as a bridge vector of WNV [52,53]. In contrast, in the United States *Cx. pipiens pipiens *feeds both on human and birds and is considered the main bridge vector of WNV Central US [52,54].

Several studies have shown the importance of migratory birds from the Sub-Saharan region as introducers of WNV into Europe [55-58], a plausible mechanism to explain the occurrence of multiple WNV outbreaks in Northern Italy. Genetic analysis of WNV isolates obtained from field collected mosquitoes during the 2008 outbreak showed, in northern Italy, a high degree (98.8%) of similarity with a virus isolate obtained from an earlier outbreak of 1998 [10,59]. Serological investigations of residential birds in the area of the 2008 outbreak showed high levels of WNV seroprevalence near the sites with high level of mosquito infection [10,17]. These two findings suggest that enzootic WNV transmission in Northern Italy may involve another mechanism (in addition to the contribution of migratory birds) for the recrudescence of WNV transmission.

The suitability maps predicted that the highest number of mosquito adults for all three species will be found in the plains where the rice fields and the main city are located. The predictions indicated that the hot-spots coincide with locations where the highest number of mosquitoes was collected. These findings indicate that the three mosquito species are abundant in the plain, underlining the importance of control program in that area. The map also showed that the Po River, one of the most important breeding habitats for local and migratory birds [19], is predicted to have the highest vector abundance. The results of this study can be used to improve the surveillance and control of WNV vectors. The IPLA Institute, responsible for the vector control interventions in eastern Piedmont, coupled the model developed in this study with weather forecasting in order to define priority areas and targeted actions to reduce vector abundance and prevent WNV establishment and amplification. A similar surveillance system based on model-derived predictions derived from environmental and climatic variable is currently used to predict the abundance of *Cx. tarsalis*, one of the main vectors of WNV in California [60]. One of the limitations of this study has been the lack of information on WNV circulation in birds and mosquitoes. Future studies will attempt to incorporate information about the composition of the bird population in the area, and the role of different bird species and the landscapes they utilize in the potential establishment of WNV in eastern Piedmont.

## Conclusions

The distribution of three putative vectors of WNV within an area of Northern Piedmont, Italy, was spatially heterogeneous and was accurately predicted after accounting for the contribution of environmental and climatic factors. The vector suitability maps derived from our study can help inform surveillance and control programmes about the location of areas that, due to their environmental suitability, could potentially become points of entry of WNV into the region. The development of carefully validated vector suitability maps can also help inform public health officers about the entomologic potential for the introduction of other vector-borne diseases of current or future global significance.

## Competing interests

The authors declare that they have no competing interests.

## Authors' contributions

All authors participated in the conceptualization of the study. DB built the GIS system and analyzed the data. DB and GVP drafted the manuscript. AM and LBalbo coordinated the mosquito collections. UK and GVP provided statistical advice. All authors participated in the revision of the manuscript and approved the submitted version.

## Supplementary Material

Additional file 1 **Weather conditions and model evaluation parameters**. File contains: table with the parameters used in model selection process, plots showing the weather conditions for the period 2000-2006, model residuals, and model performance. Click here for file

## References

[B1] Diamond MS West Nile Encephalitis Virus Infection: Viral Pathogenesis and the Host Immune Response 2008 1 Springer

[B2] Sfakianos JN West Nile Virus 2004 Chelsea House Publications

[B3] Kramer LD Styer LM Ebel GD A Global Perspective on the Epidemiology of West Nile Virus Annu Rev Entomol 2008 53 61 81 10.1146/annurev.ento.53.103106.093258 17645411

[B4] Smithburn KC Differentiation of the West Nile Virus from the Viruses of St. Louis and Japanese B Encephalitis J Immunol 1942 44 25 31

[B5] Calistri P Giovannini A Hubalek Z Ionescu A Monaco F Savini G Lelli R Epidemiology of West Nile in Europe and in the Mediterranean Basin Open Virol J 2010 4 29 37 20517490 10.2174/1874357901004010029 PMC2878979

[B6] Murray KO Mertens E Despres P West Nile virus and its emergence in the United States of America Vet Res 2010 41 67 10.1051/vetres/2010039 21188801 PMC2913730

[B7] Joubert L Oudar J Hannoun C Beytout D Corniou B Guillon JC Panthier R Epidemiology of the West Nile virus: study of a focus in Camargue. IV. Meningo-encephalomyelitis of the horse Ann Inst Pasteur (Paris) 1970 118 239 247 5461277

[B8] Hubálek Z Mosquito-borne viruses in Europe Parasitol Res 2008 103 Suppl 1 S29 43 19030884 10.1007/s00436-008-1064-7

[B9] Filipe AR Isolation in Portugal of West Nile virus from *Anopheles maculipennis *mosquitoes Acta Virol 1972 16 361 4403183

[B10] Monaco F Lelli R Teodori L Pinoni C Di Gennaro A Polci A Calistri P Savini G Re-emergence of West Nile virus in Italy Zoonoses Public Health 2010 57 476 486 10.1111/j.1863-2378.2009.01245.x 19638165

[B11] Fyodorova MV Savage HM Lopatina JV Bulgakova TA Ivanitsky AV Platonova OV Platonov AE Evaluation of potential West Nile virus vectors in Volgograd region, 2003 (Diptera: *Culicidae*): species composition, bloodmeal host utilization, and virus infection rates of mosquitoes J Med Entomol 2006 43 552 563 10.1603/0022-2585(2006)43[552:EOPWNV]2.0.CO;2 16739415

[B12] Balenghien T Vazeille M Grandadam M Schaffner F Zeller H Reiter P Sabatier P Fouque F Bicout DJ Vector competence of some French *Culex *and *Aedes *mosquitoes for West Nile virus Vector Borne Zoonotic Dis 2008 8 589 595 10.1089/vbz.2007.0266 18447623

[B13] Almeida APG Galão RP Sousa CA Novo MT Parreira R Pinto J Piedade J Esteves A Potential mosquito vectors of arboviruses in Portugal: species, distribution, abundance and West Nile infection Trans R Soc Trop Med Hyg 2008 102 823 832 10.1016/j.trstmh.2008.03.011 18455742

[B14] Autorino GL Battisti A Deubel V Ferrari G Forletta R Giovannini A Lelli R Murri S Scicluna MT West Nile virus epidemic in horses, Tuscany region, Italy Emerging Infect Dis 2002 8 1372 1378 12498650 10.3201/eid0812.020234 PMC2738505

[B15] Romi R Pontuale G CIufolini MG Fiorentini G Marchi A Nicoletti L Cocchi M Tamburro A Potential vectors of West Nile virus following an equine disease outbreak in Italy Med Vet Entomol 2004 18 14 19 10.1111/j.1365-2915.2004.0478.x 15009441

[B16] Rizzoli A Rosà R Rosso F Buckley A Gould E West Nile virus circulation detected in northern Italy in sentinel chickens Vector Borne Zoonotic Dis 2007 7 411 417 10.1089/vbz.2006.0626 17767411

[B17] Calistri P Giovannini A Savini G Monaco F Bonfanti L Ceolin C Terregino C Tamba M Cordioli P Lelli R West Nile virus transmission in 2008 in north-eastern Italy Zoonoses Public Health 2010 57 211 219 10.1111/j.1863-2378.2009.01303.x 20042066

[B18] Macini P Squintani G Finarelli AC Angelini P Martini E Tamba M Dottori M Bellini R Santi A Loli Piccolomini L Po C Detection of West Nile virus infection in horses, Italy, September 2008 Euro Surveill 2008 13 pii = 18990 18822243

[B19] Spangesi M Serra L Uccelli d'Italia 2005 Quad. Cons. Natura, 22, Min. Ambiente - Ist. Naz. Fauna Selvatica

[B20] Barzon L Franchin E Squarzon L Lavezzo E Toppo S Martello T Bressan S Pagni S Cattai M Piazza A Pacenti M Cusinato R Palù G Genome sequence analysis of the first human West Nile virus isolated in Italy in 2009 Euro Surveill 2009 14 pii = 19384 19941775

[B21] Busani L Capelli G Cecchinato M Lorenzetto M Savini G Terregino C Vio P Bonfanti L Pozza MD Marangon S West Nile virus circulation in Veneto region in 2008-2009 Epidemiol Infect 2010 818 825 10.1017/S0950268810001871 20670469

[B22] Istituto Zooprofilattico Sperimentale di Teramo http://sorveglianza.izs.i

[B23] Calistri P Monaco F Savini G Guercio A Purpari G Vicari D Cascio S Lelli R Further spread of West Nile virus in Italy Vet Ital 2010 46 467 474 21120802

[B24] Stojanovic J Scott HG Mosquitoes of the Italian Biogeographical Area Which Includes the Republic of Malta, the French Island of Corsica and All of Italy Except the Far Northern Provinces 1997

[B25] Allen RA Wilkes WW Lewis CN Meisch MV Riceland mosquito management practices for *Anopheles quadrimaculatus *larvae J Am Mosq Control Assoc 2008 24 534 537 10.2987/5792.1 19181061

[B26] Margalit J Dean D The story of *Bacillus thuringiensis *var. *israelensis *(B.t.i.) J Am Mosq Control Assoc 1985 1 1 7 2906651

[B27] Neteler M Mitasova H Open Source GIS: A GRASS GIS Approach 2004 2 Springer

[B28] NASA MODIS Website http://modis.gsfc.nasa.gov/

[B29] Arpa Piemonte http://www.arpa.piemonte.it/

[B30] European Environment Agency http://www.eea.europa.eu/

[B31] Ripley BD Spatial statistics 1981 Wiley

[B32] Getis A Ord JK The Analysis of Spatial Association by Use of Distance Statistics Geographical Analysis 1992 24 189 206

[B33] Zuur AF Ieno EN Walker NJ Saveliev AA Smith GM Mixed effects models and extensions in ecology with R 2009 Springer

[B34] Rue H Martino S Chopin N Approximate Bayesian inference for latent Gaussian models by using integrated nested Laplace approximations J Roy Statistical Society: Series B (Statistical Methodology) 2009 71 319 392 10.1111/j.1467-9868.2008.00700.x

[B35] Besag J York J Mollié A Bayesian image restoration, with two applications in spatial statistics Ann I Stat Math 1991 43 1 59 10.1007/BF00116466

[B36] Rue Ha Martino S Approximate Bayesian inference for hierarchical Gaussian Markov random field models J Statist Plann Inference 2007 137 3177 3192 10.1016/j.jspi.2006.07.016

[B37] Schrödle B Held L Riebler A Danuser J Using integrated nested Laplace approximations for the evaluation of veterinary surveillance data from Switzerland: a case-study J Roy Statistical Society: Series C (Applied Statistics) 2011 60 261 279 10.1111/j.1467-9876.2010.00740.x

[B38] Spiegelhalter DJ Best NG Carlin BP van der Linde A Bayesian measures of model complexity and fit J Roy Statistical Society: Series B (Statistical Methodology) 2002 64 583 639 10.1111/1467-9868.00353

[B39] Wilberg MJ Bence JR Performance of deviance information criterion model selection in statistical catch-at-age analysis Fish Res 2008 93 212 221 10.1016/j.fishres.2008.04.010

[B40] Gneiting T Balabdaoui F Raftery AE Probabilistic forecasts, calibration and sharpness J Roy Statistical Society: Series B (Statistical Methodology) 2007 69 243 268 10.1111/j.1467-9868.2007.00587.x

[B41] R Development Core Team 2011 R Foundation for Statistical Computing, Vienna, Austria

[B42] Balenghien T Carron A Sinègre G Bicout DJ Mosquito density forecast from flooding: population dynamics model for *Aedes caspius *(Pallas) Bull Entomol Res 2010 100 247 254 10.1017/S0007485309990745 20170592

[B43] Cailly P Balenghien T Ezanno P Fontenille D Toty C Tran A Role of the repartition of wetland breeding sites on the spatial distribution of Anopheles and Culex, human disease vectors in Southern France Parasit Vectors 2011 6 65 10.1186/1756-3305-4-65 PMC3114004 21548912

[B44] Ponçon N Balenghien T Toty C Baptiste Ferré J Thomas C Dervieux A L'ambert G Schaffner F Bardin O Fontenille D Effects of local anthropogenic changes on potential malaria vector *Anopheles hyrcanus *and West Nile virus vector *Culex modestus*, Camargue, France Emerging Infect Dis 2007 13 1810 1815 18258028 10.3201/eid1312.070730 PMC2876767

[B45] Del Giudice P Schuffenecker I Vandenbos F Counillon E Zellet H Human West Nile virus, France Emerging Infect Dis 2004 10 1885 1886 15515250 10.3201/eid1010.031021 PMC3323251

[B46] Durand B Chevalier V Pouillot R Labie J Marendat I Murgue B Zeller H Zientara S West Nile Virus Outbreak in Horses, Southern France, 2000: Results of a Serosurvey Emerg Infect Dis 2002 8 777 782 12141961 10.3201/eid0808.010486 PMC2732513

[B47] Balenghien T Fouque F Sabatier P Bicout DJ Horse-, bird-, and human-seeking behavior and seasonal abundance of mosquitoes in a West Nile virus focus of southern France J Med Entomol 2006 43 936 946 10.1603/0022-2585(2006)43[936:HBAHBA]2.0.CO;2 17017231

[B48] Fasola M Ruiz X The Value of Rice Fields as Substitutes for Natural Wetlands for Waterbirds in the Mediterranean Region Colonial Waterbirds 1996 19 122 128

[B49] Zeller HG Schuffenecker I West Nile virus: an overview of its spread in Europe and the Mediterranean basin in contrast to its spread in the Americas Eur J Clin Microbiol Infect Dis 2004 23 147 156 10.1007/s10096-003-1085-1 14986160

[B50] Orshan L Bin H Schnur H Kaufman A Valinsky A Shulman L Weiss L Mendelson E Pener H Mosquito vectors of West Nile Fever in Israel J Med Entomol 2008 45 939 947 10.1603/0022-2585(2008)45[939:MVOWNF]2.0.CO;2 18826039

[B51] Trawinski PR Mackay DS Identification of environmental covariates of West Nile virus vector mosquito population abundance Vector Borne Zoonotic Dis 2010 10 515 526 10.1089/vbz.2008.0063 20482343

[B52] Fonseca DM Keyghobadi N Malcolm CA Mehmet C Schaffner F Mogi M Fleischer RC Wilkerson RC Emerging vectors in the *Culex pipiens *complex Science 2004 303 1535 1538 10.1126/science.1094247 15001783

[B53] Vinogradova EB Shaĭkevich EV Differentiation between the urban mosquito *Culex pipiens pipiens *F. *molestus *and *Culex torrentium *(Diptera: Culicidae) by the molecular genetic methods Parazitologia 2005 39 574 576 16396396

[B54] Hamer GL Kitron UD Brawn JD Loss SR Ruiz MO Goldberg TL Walker ED *Culex pipiens*(Diptera: *Culicidae*): a bridge vector of West Nile virus to humans J Med Entomol 2008 45 125 128 10.1603/0022-2585(2008)45[125:CPDCAB]2.0.CO;2 18283952

[B55] Pfefer M Dobler G Emergence of zoonotic arboviruses by animal trade and migration Parasit Vectors 2010 3 35 10.1186/1756-3305-3-35 20377873 PMC2868497

[B56] Figuerola J Soriguer R Rojo G Gómez Tejedor C Jimenez-Clavero MA Seroconversion in wild birds and local circulation of West Nile virus, Spain Emerging Infect Dis 2007 13 1915 1917 18258046 10.3201/eid1312.070343 PMC2876749

[B57] Jourdain E Toussaint Y Leblond A Bicout DJ Sabatier P Gauthier-Clerc M Bird species potentially involved in introduction, amplification, and spread of West Nile virus in a Mediterranean wetland, the Camargue (Southern France) Vector Borne Zoonotic Dis 2007 7 15 33 10.1089/vbz.2006.0543 17417954

[B58] Linke S Ellerbrok H Niedrig M Nitsche A Pauli G Detection of West Nile virus lineages 1 and 2 by real-time PCR J Virol Methods 2007 146 355 358 10.1016/j.jviromet.2007.05.021 17604132

[B59] Savini G Monaco F Calistri P Lelli R Phylogenetic analysis of West Nile virus isolated in Italy in 2008 Euro Surveill 2008 13 pii = 19048 19040827

[B60] Barker CM Reisen WK Kramer VL California State Mosquito-borne Virus Surveillance and Response Plan: a retrospective evaluation using conditional simulations Am J Trop Med Hyg 2003 68 508 518 12812335 10.4269/ajtmh.2003.68.508

