# Supplementary material for: Spatio-temporal patterns of distribution of West Nile virus vectors in eastern Piedmont Region, Italy
Source: Parasit Vectors. 2011 Dec 9;4:230. doi: 10.1186/1756-3305-4-230 (PMC3251540; doi:10.1186/1756-3305-4-230)
Supplement: Additional file 1 — Weather conditions and model evaluation parameters. File contains: table with the parameters used in model selection process, plots showing the weather conditions for the period 2000-2006, model residuals, and model performance. [file 1756-3305-4-230-S1.PDF]

Additional file 1

Table 1-A. Top three models predicting the weekly median number of *Oc. caspius*, *Cx. pipiens* and *Cx. modestus* per trap. DIC= Deviance Information Criterion; LS= Logarithmic Score.

	Predictor								
	**DISTR**	**DISTU**	**ELEV**	**RAIN**	**TWEEK**	**NDVI**	**SIN**	**DIC**	**LS**
Oc. caspius	-0.034	-	-0.006	0.008*	0.092*	0.789*	0.853*	1646.8	0.68
	-0.03	-	-0.005	-	0.072*	-0.151	0.912*	1657.3	1.05
	-0.015	-	-0.004	-	0.083*	-	0.856*	1678.2	1.23
Cx. pipiens	-	-0.122	-0.014*	0.018*	0.070*	-	0.278*	1434.5	0.75
	-0.012	-0.051	-0.009	-0.001	0.056*	-	0.301*	1447.2	1.23
	-0.008	-0.042	-0.011*	0.002*	0.061*	-0.123	0.245*	1452.4	1.55
Cx. modestus	-0.081	-	-0.021*	-	0.083*	0.864*	0.589*	2114.3	1.11
	-0.067	-0.012	-0.034*	-	0.078*	0.564*	0.678*	2125.2	1.56
	-0.067	-	-0.034*	-0.013	0.055*	0.254	0.765*	2144.7	1.78

*P < 0.05.

DISTR= distance from rice fields; DISTU= distance from urban area; ELEV= elevation of trapping location; RAIN= cumulative rainfall (mm) for the 10 days before the collection; TWEEK= weekly mean temperature for the week before collection; NDVI= NDVI value of the pixel of trapping location; SIN= sinusoidal function representing the seasonality (1=1st week of August); RNDTRAP= unstructured random effect for trapping locations; SRNDTRAP= spatial structured random effect for trapping locations.

Figure 1-A. Weekly patterns of rainfall and temperature for the years 2000 to 2006 estimated from three weather stations scattered throughout the study area. Blue bars indicate the comulative weekly rainfall (in mm) and black lines 7-d moving averages of daily mean temperature (in °C).

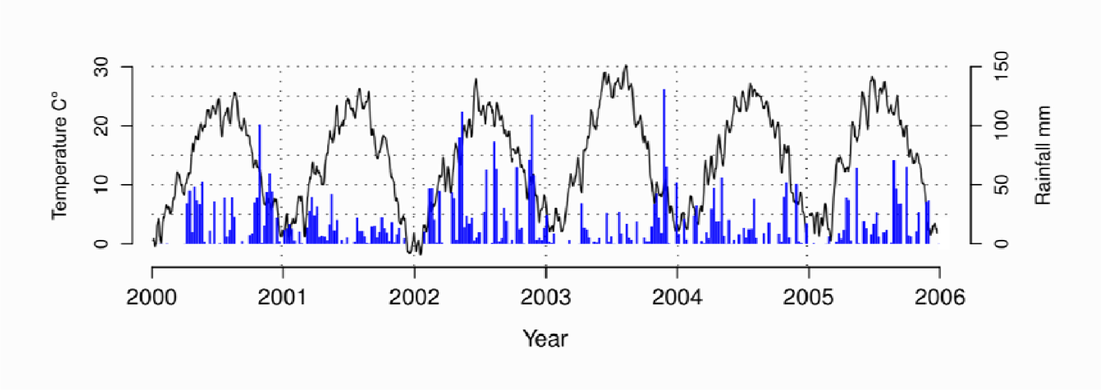

Figure 2-A. Standardized residuals calculated from the best fit model for *Oc. Caspius* (A), *Cx. pipiens* (B), and *Cx. modestus* (C).

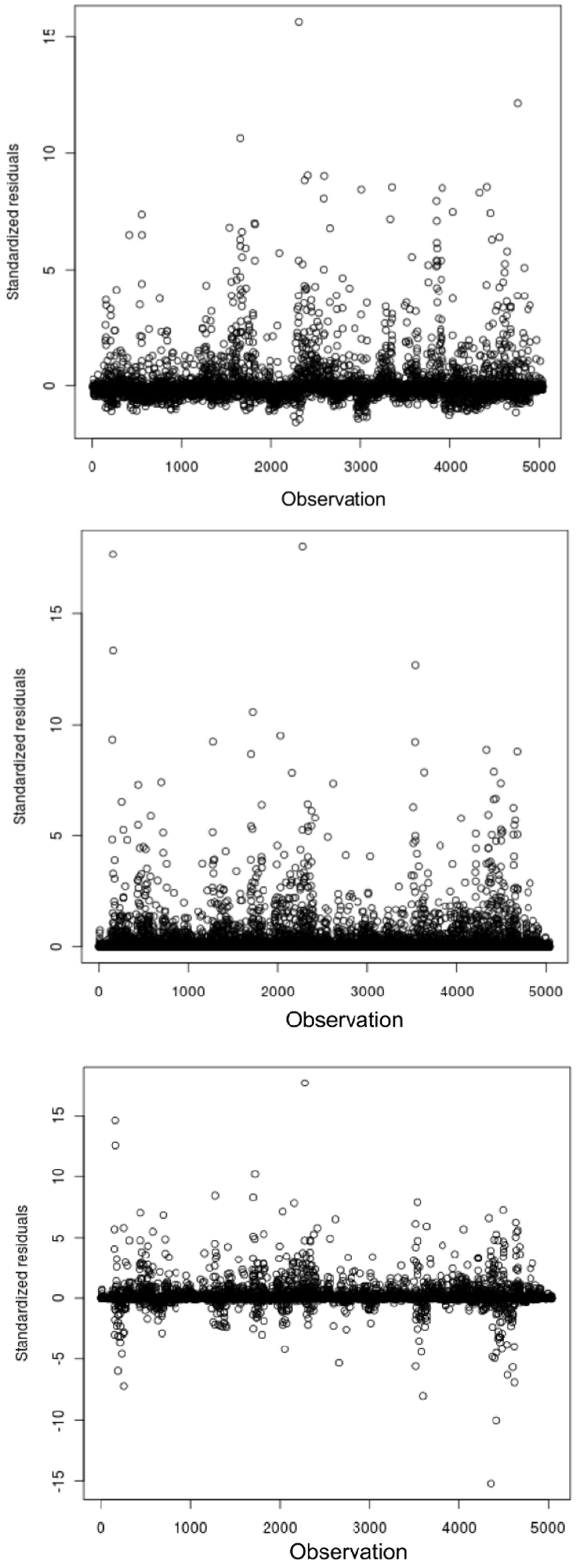

Figure 3-A. Observed (black line) and predicted (red line) number of *Oc. caspius* per trap for the year 2010 estimated at six randomly selected trap locations.

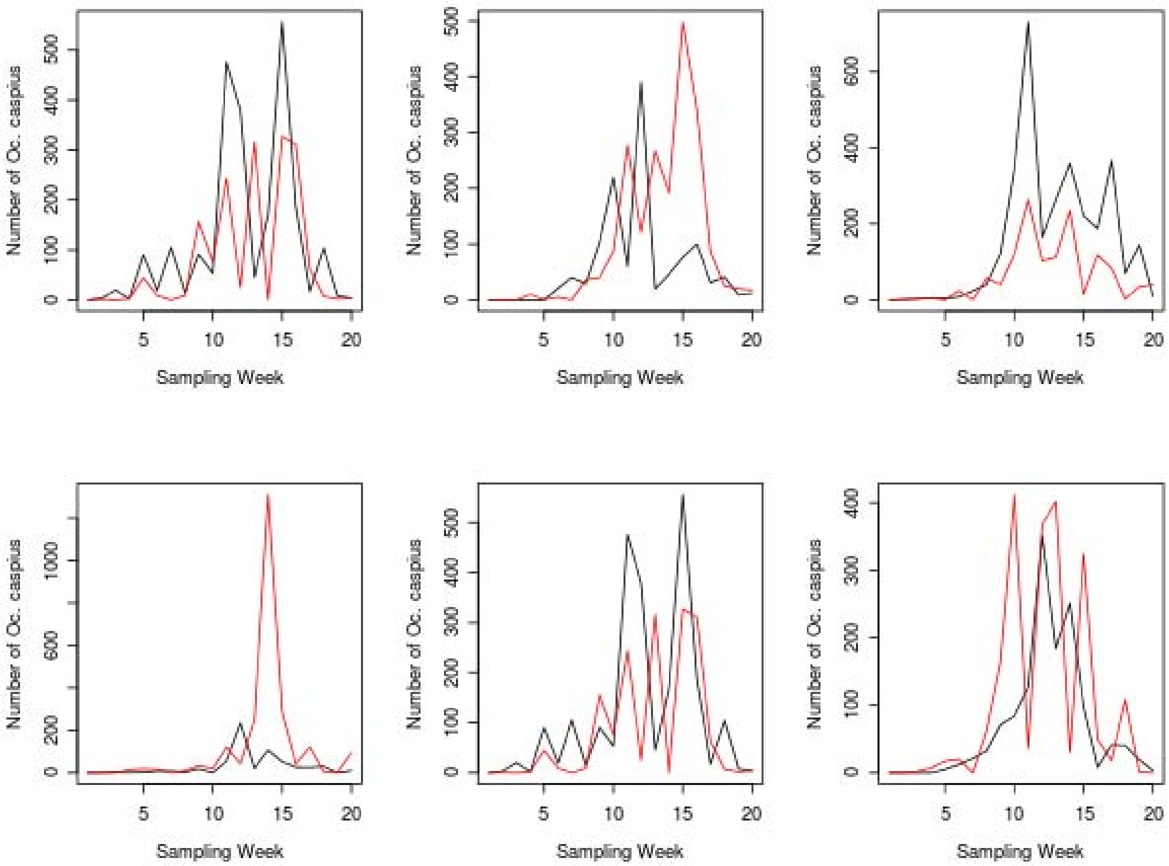

Figure 4-A. Observed (black line) and predicted (red line) number of *Cx. pipiens* per trap for the year 2010 estimated at six randomly selected trap locations.

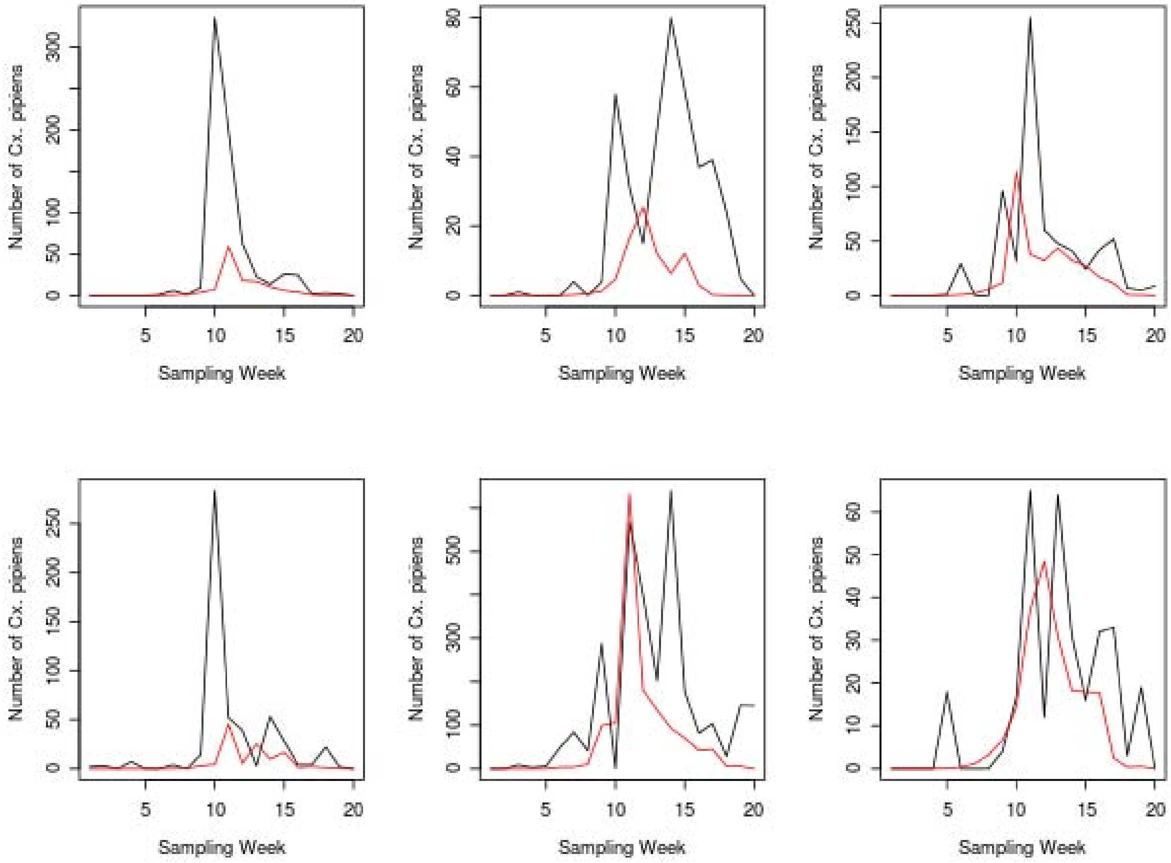

Figure 5-A. Observed (black line) and predicted (red line) number of *Cx. modestus* per trap for the year 2010 estimated at six randomly selected trap locations.

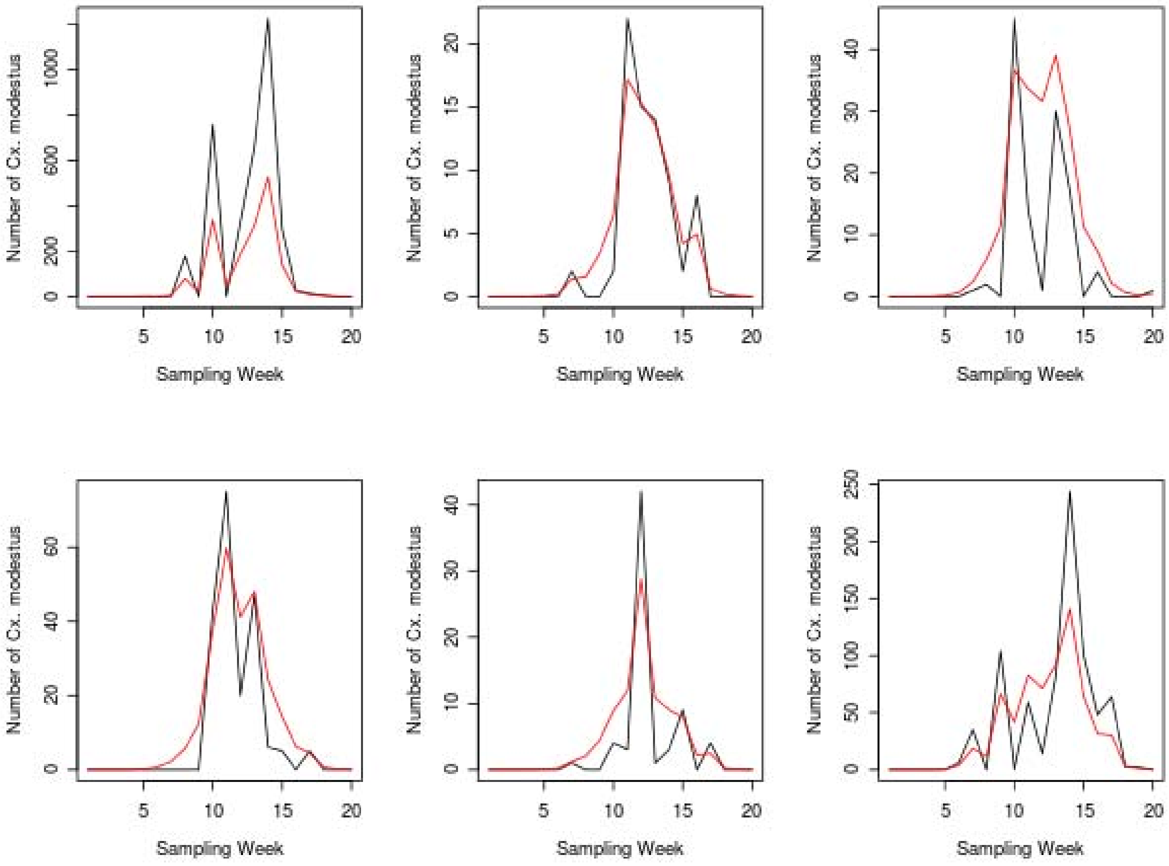

Table 2-A. Full code used to run the GLMM models with Integrated Nested Laplace Approximations (INLA).

```
full_model_formula <- mosquito_count ~ RAIN + TWEEK + SIN + ELEV + NDVI + DISTU
+ DISTR + DISTW + RICEA + f(TRAP_ID, model="besag", graph.file ="spatial.graph")  +
f(TRAP_ID,model="iid")

full_model <-
inla(full_model_formula,data=mosquito_data,family="zeroinflatednbinomial",control.comp
ute=list(dic=T,mlik=T,cpo=T))
```
